# Apoptosis induction in acute promyelocytic leukemia cells through upregulation of *CEBPα *by miR-182 blockage

**DOI:** 10.22099/mbrc.2018.27625.1297

**Published:** 2018-03

**Authors:** Mohammadreza Sharifi, Mahdi Fasihi-Ramandi, Abdolkarim Sheikhi, Abbas Moridnia, Maryam Saneipour

**Affiliations:** 1 Department of Genetics and Molecular Biology, School of Medicine, Isfahan University of Medical Science, Isfahan, Iran; 2 Molecular Biology Research Center, Baqiyatallah University of MedicalSciences, Tehran, Iran; 3 Department of Immunology, School of Medicine, Dezful University of Medical Science, Dezful, Iran

**Keywords:** miR-182-5p, Locked Nucleic Acid, Acute Promyelocytic Leukemia, CEBPα

## Abstract

MicroRNAs (miRNAs) involved in regulation of the genes. The CCAAT/enhancer-binding protein-α (*CEBPα*) is a crucial transcription factor for normal hematopoiesis and cell cycle that frequently disrupted in human acute myeloid leukemia (AML). The miR-182 up-regulation in several malignant diseases such as AML was reported, in the other hand bioinformatics analysis revealed *CEBPα* targeted by miR-182.miR-182-5p inhibition in human acute promyelocytic leukemia (APL) cell line was performed by using locked nucleic acid (LNA) and subsequently miR-182-5p and *CEBPα *expression, apoptosis, necrosis and cell proliferation were measured. After LNA-anti-miR-182-5p transfection to cells at different time points, miR-182-5p down regulation and *CEBPα* overexpression was revealed in the LNA-anti-miR group compared to the control groups. The cell viability was meaningfully varied between LNA-anti-miR and control groups. Increasing of the apoptotic ratio was linked to miR-182-5p inhibition in the LNA-anti-miR group rather than other groups. Similarly, the necrotic ratio in the LNA-anti-miR group was higher.Our results supported the hypothesis that miR-182-5p inhibition can reduce the cell viability predominantly due to induces apoptosis and necrosis. The present results can apply in translational medicine for investigation of antisense therapy and drug development in leukemia.

## INTRODUCTION

Acute promyelocytic leukemia (APL) is one of the severe forms of acute leukemia with a very fast clinical signs start, poor response to chemotherapy treatment and disseminated intravascular coagulation that is characterized by high mortality rates [1]. This leukemia mainly causes due to translocation between retinoic acid receptor alpha (RARα) gene and acid promyelocytic leukemia (PML) gene [2-4]. Hematopoiesis is an extremely arranged interaction of lineage-specific transcription factors that moving pluripotent precursor cells to the differentiated mature blood cells [5]. The evidences suggests that this differentiation besides the various hematopoietic lineages is somewhat regulated by microRNAs (miRNAs) [6]. Thus, expression of definite transcription factors was regulated by miRNAs in post-transcriptional level [7]. Hematopoietic transcription factors or miRNAs deregulation is a common episode in the molecular pathogenesis process of human leukemias [8]. The CCAAT/enhancer-binding protein-α (*CEBPα*) is a crucial transcription factor for normal hematopoiesis that frequently disrupted in human acute myeloid leukemia (AML). The *CEBPα* gene has a vital role for myeloid differentiation towards mature granulocytes [9]. The various *CEBPα* downstream effectors during the normal hematopoiesis including miR-223 have been defined [10]. The potentially anti-proliferative effect of *CEBPα* in myeloid cells recommended that it maybe function as a suppressor for leukemogenesis [11]. Pabst *et al*, represented that 7.3% of AML patients carried spontaneous *CEBPα* mutations in the tumor cell DNA [12].

 MiRNAs are short non-coding RNA, about 19-25 nucleotides and play an important role in biological processes such as cell cycle, differentiation, growth, metabolism, aging and apoptosis. MiRNAs plays a key role in a wide range of diseases including cardiovascular diseases, rheumatologic, infectious, inflammatory, autoimmune, metabolic and cancer [13]. Up to now, the role of miRNAs in a number of cancers has been shown, for example, overexpression of miR-21 in breast cancer and miR-145 downregulation in lung cancer was reported. The miR-183/96/182 cluster is a highly conserved miRNA cluster that their members are located on 7q32.2 human chromosome that is abnormally expressed in a variety of tumors [14]. MiR-182 belongs to the miR-183-96-182 cluster that plays a vital role in tumorgenesis and cancer development [14]. Among these miRNAs cluster, miR-182 is an oncogene and their oncogenic features confirmed by negative regulation of multiple tumor suppressor genes including *HMGA2*, *BRCA1*, *FOXO3*, and *MTSS1* [15-18]. The overexpression of miR-182 in breast cancer, melanoma, lung, colon and prostate cancer have been reported [19, 20]. Up-regulation of miR-182 in Chronic lymphocytic leukemia (CLL) [21] and in APL was described [22]. In the present study using the bioinformatics softwares include miRanda, miRDB, miRWalk and Targetscan predicted that *CEBPα* targeted by miR-182. Also, previous described the *CEBPα* gene is a one of a target gene of miR-182 [23]. 

 According to these evidences, we suggested inhibition of miR-182-5p has been anti-proliferation effect on APL through regulation of *CEBPα* gene. In the present study, miR-182-5p inhibition was done by LNA to assess their effect on cell proliferation and apoptosis through regulation of *CEBPα* gene in APL cell line (HL-60). 

## MATERIALS AND METHODS

This study was approved by the local ethics committee of Isfahan University of Medical Sciences (IRAN) and the studies have been approved by the appropriate institutional and/or a national research ethics committee and have been performed in accordance with the ethical standards as laid down in the 1964 Declaration of Helsinki and its later amendments or comparable ethical standards.

**Cell culture** **: **HL-60 cell line (human APL cell line) was purchased from National Cell Bank of Iran (Pasteur Institute, Tehran, Iran). Cell culture was sustained at RPMI 1640 medium (Gibco, Paisley, UK). This media was supplemented with fetal bovine serum (FBS; Gibco) 10% v/v, GM-CSF 10 ng/ml (R&D Systems, Minneapolis, MN, USA), 100 U/ml of penicillin and 100 μg/ml of streptomycin (Sigma-Aldrich, Saint Louis, MO, USA) in 25-cm^2^ culture flasks with 5% CO_2_ (Nunc, Roskilde, Denmark) at 37°C. To maintain cells in exponential phase, the cells were passaged twice a week.

**Cell transfection: **Nucleotide sequences of miR-182-5p were achieved from www.mirbase.org as 5′-UUU GGC AAU GGU AGA ACU CAC ACU-3′ (MIMAT 0000259). MicroRNA inhibitor negative control (scrambled) and miRCURY LNA microRNA Inhibitor for hsa-miR-182-5p was purchased from Exiqon (Copenhagen, Denmark). LNA anti-miR and scrambled oligonucleotides have been labeled at the 5´ end with a fluorescent dye, 6-FAM. HL-60 cells transfection was performed by PolyFect™ transfection reagent kit (Qiagen, Hilden, Germany) according to previous studies [22, 24].

**Reverse transcriptase microRNA real-time PCR** **:** To determination efficiency of miR-182-5p inhibition by LNA-anti-miR, qRT-PCR were done. In the 24, 48 and 72 h after transfection, total RNA extracted by miRCURY RNA Isolation Kit (Exiqon, Copenhagen, Denmark), and then cDNA synthesized by Universal cDNA Synthesis Kit (Exiqon, Copenhagen, Denmark). The qPCR by SYBR Green Master Mix Kit was done (Exiqon, Copenhagen, Denmark) and specific primers for miR-182-5p obtained from Exiqon. For qPCR tests was used ABI Step One Plus (Applied Biosystems, Foster City, CA, USA) instrument and ΔΔCt method for data analysis.

***CEBPα*** **gene qRT-PCR:** The expression level of *CEBPα* gene was determined by qRT-PCR. The miRCURY RNA Isolation Kit and Universal cDNA Synthesis Kit from Exiqon (Copenhagen, Denmark) were used for total RNA extraction and cDNA synthesis. The SYBR Green Master Mix Kit (Exiqon, Copenhagen, Denmark) was used for qRT-PCR and *GAPDH* gene act as an internal control. The primers were used in this study for *CEBPα* include F: 5'-CTA GAG ATC TGG CTG TGG GG-3' and R: 5'-TCT GGG ATG GAC TGA TCG TG-3', and for *GAPDH* include F: 5'-GGT GTG AAC CAT GAG AAG TAT GA-3' and R: 5'-GAG TCC TTC CAC GAT ACC AAA G-3'. The Step One Plus^TM^ (Applied Biosystems, Foster City, CA, USA) instrument and ΔΔCt method were used for data analysis in real-time PCR tests.

**Cell viability assay: **The MTT (3-[4, 5 dimethylthiazol-2-yl]-2, 5-diphenyl tetrazolium bromide) assay was performed to assessed of cell viability. In this method, MTT is a reduction by mitochondrial dehydrogenase in live cells to produce purple formazan. The alteration is directly associated with the number of living cells. MTT assay was done at 24, 48 and 72 h after transfection in HL-60 cells according to previous studies [24, 25]. 

**Apoptosis and necrosis assay:** Apoptosis and necrosis detected by FITC Annexin-V apoptosis detection Kit with PI (Bio legend, San Diego, USA) in HL-60 cells. Annexin-V detected phosphatidylserine on apoptotic cells. Propidium iodide (PI) staining was used for detection of necrotic cells (untreated cells were used for control). The apoptosis and necrosis assay at 24, 48 and 72 h after transfection was done according to the manufacturer’s instructions and the cells were evaluated by FACS Calibur Flow Cytometer (BD, California, USA). 

**Data analysis: **All experiments were repeated three times. The results were analysis by SPSS version 20 (IBM, New York, NY, USA) software. Two-way ANOVA (two-way analysis of variance) also post hoc test to examine were considered. Data presented as mean ± SD. Statistical significance was defined as *p*<0.05.

## RESULTS

**Figure 1:**
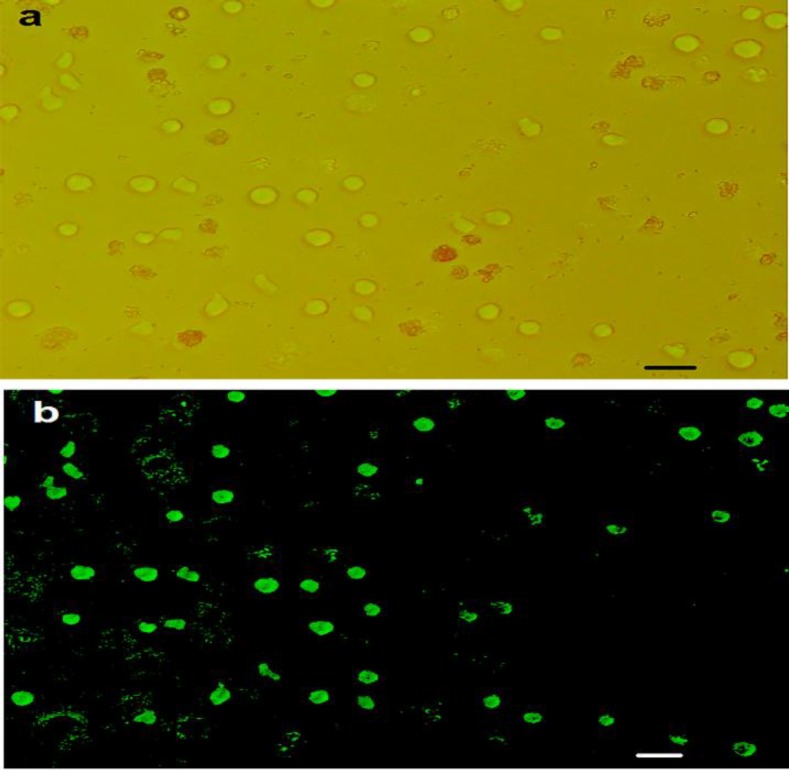
Cells were transfected with 6-FAM fluorescein-conjugated LNA-anti-miR-182-5p and then evaluated the transfection efficacy; they were detected by fluorescent microscope Phase contrast (**a**) and fluorescent (**b**). Pictures of the same field displayed majority of the cells are transfected. Scale bar 50µm

**Figure 2:**
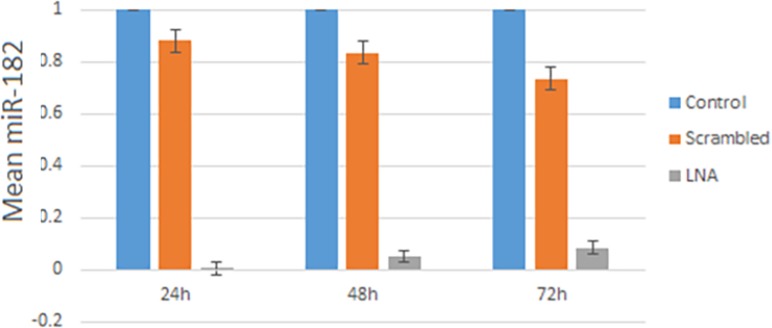
miR-182 expression level by qRT PCR at 24, 48 and 72 h after transfection; data analysis performed by the ΔΔCt method and untreated group (control) was considered as a reference. Data are mean ±S.D. of triple independent experiments

Since the oligonucleotides have been fluorescence-conjugated, transfection efficiency determined by fluorescence microscopy. Transfection efficiency was approximately 90% (Fig. 1). The miR-182-5p expression was considered by qRT-PCR in transfected group with LNA-anti-miR, scrambled and untreated groups at three-time points 24, 48 and 72 h after transfection. The expression of miR-182-5p was noticeably lower in the LNA-anti-miR group compared with the control groups (*P*<0.025). MiR-182-5p expression at 24 h after transfection was at the lowest level and steadily increased at the next time points (Fig. 2). 

**Figure 3:**
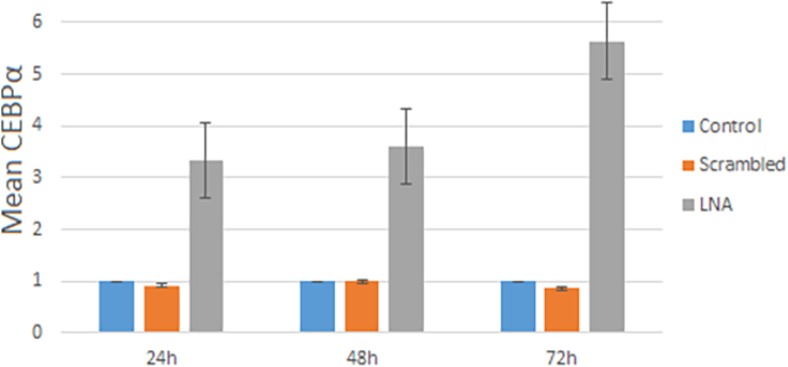
Expression level of *CEBPα* by qRT PCR at 24, 48 and 72 h after transfection; data analysis performed by the ΔΔCt method and untreated group (control) was considered as a reference. Data are mean ±S.D. of triple independent experiments. *P*< 0.021

Expression level of *CEBPα* gene was considered in HL-60 cells include LNA-anti-miR group, scrambled and untreated groups by qRT-PCR at three-time points 24, 48, and 72 h after transfection. In the LNA-anti-miR group the *CEBPα* expression after transfection was increased rather than untreated groups (p<0.021). The upper-level expression of *CEBPα* was at 72 h after transfection in HL-60 cells (Fig. 3).

**Figure 4:**
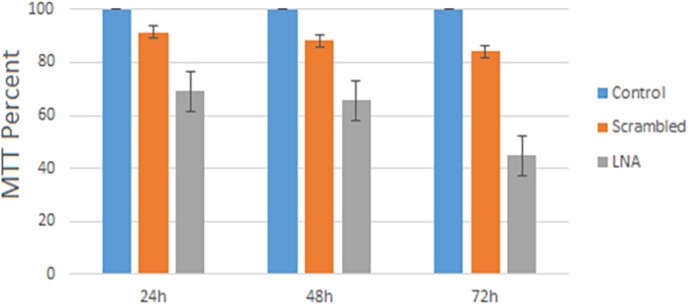
Cell viability was assessed by MTT assay. Viability in control cells at all three-time points has been considered as 100% and in other groups is accessible as a percentage of control cells. Data are mean±S.D. of triple independent experiments

The MTT assay results revealed cell viability meaningfully decreased in HL-60 cells after LNA-anti-miR transfection. The cell viability in LNA-anti-miR group of HL-60 cells steadily increased over time. The difference in cell viability was statistically significant between LNA-anti-miR group compared with scrambled and untreated groups (*P*>0.024) in 24, 48 and 72 h after transfection (Fig. 4). 

**Figure 5:**
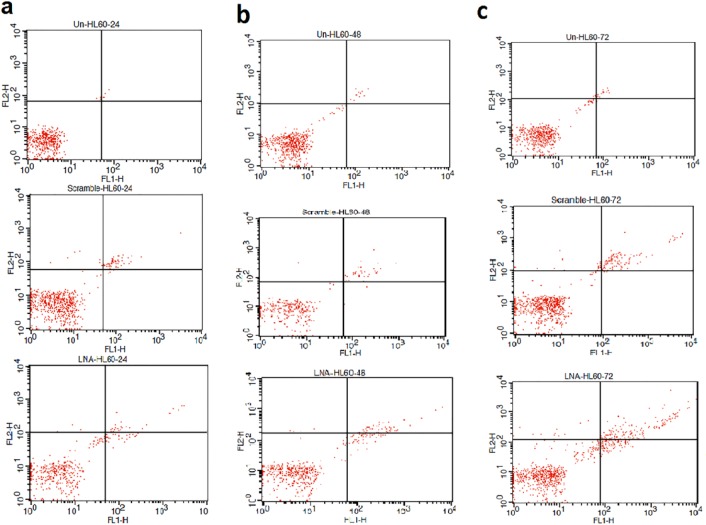
Apoptosis and necrosis assay were performed by annexin-V-PI staining at 24, 48 and 72 h after transfection. Cytofluorometric graphs are shown (**a**,**b** and **c**

**Figure 6:**
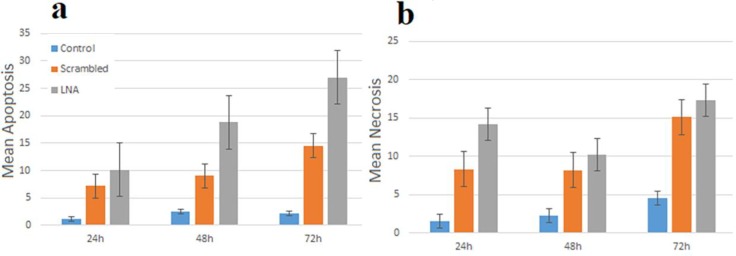
Evaluation of Apoptosis was performed by Annexin V-PI staining at 24, 48, and 72 h after transfection (**a**). Data displayed in the graph are mean ± SD of triple independent experiments. *P*< 0.028. Evaluation of necrosis was performed by Annexin V-PI staining at 24, 48, and 72 h after transfection (**b**). Data displayed in the graph are mean ± SD of triple independent experiments

Apoptosis dramatically increased in HL-60 cells transfected by LNA-anti-miR and miR-182-5p inhibition linked with apoptosis as higher than in the LNA-anti-miR group compared to another groups at three-time points (Figure 5, 6 a;* P*< 0.028). Also, necrosis significantly increased in HL-60 cells transfected by LNA-anti-miR compared to another groups at three-time points (Figure 5, 6 b;* P*< 0.03). 

## DISCUSSION

The family transcription factors of CCAAT/enhancer-binding protein (*CEBP*) shows an important role in cell proliferation and differentiation. The *CEBPα* has a predominantly regulatory effect on cell-cycle exit and induced in terminally differentiating myeloid and adipocytes cells and also activates differentiation-specific genes. The growth-inhibiting action of *CEBPα* suppresses the tumorigenesis processing in myeloid cells and probably other tissues. Also, *CEBPα* act as a member of p53-regulated growth arrest response that produced by DNA damage [11]. The expression forced of *CEBP*α obligates 3T3-L1 pre-adipoblasts to adipocyte differentiation and induces cell-cycle arrest [26], and also the antiproliferation activity of *CEBP*α encompasses to hepatocytes and Saos2 osteosarcoma cells have been described [27]. 

The role of miRNAs in cancers and especially deregulation of miRNAs in human leukemias has been described [28], for example up-regulation of miR-182 in Chronic lymphocytic leukemia (CLL) [21] and in APL was described [22]. Overexpression of miR-92a targeting *FBXW7* in cervical cancer cells and increased proliferation and tumorgenesis [29]. miR-503 and miR-182 in colon cancer cells were overexpressed and increased proliferation [30]. *FOXO1* is a target of miR-182 that is a transcription factor for regulating genes involved in apoptosis, cell cycle and metabolism. Decreased expression of this transcription factor by overexpression of miR-182 in the breast cancer causes a disturbance in apoptosis [31]. Up-regulation of the miR-183/96/182 cluster is associated with metastatic features in hepatocellular carcinoma [32]. Up to now, several miRNAs reported as pro-metastatic such as miR-10b, miR-373, miR-520c, miR-21, miR-143 and miR-182 [33]. Up-regulation of miR-189, miR-21 and also downregulation of let-7 family in breast cancer were described [34]. 

In this study we used LNA-anti-miR for silencing of miR-182 in APL cell line. Nearly complete miR-182-5p downregulation was confirmed after LNA-anti-miR transfection by qRT-PCR. The decrease of cell viability revealed associated with miR-182-5p blockage through regulation of *CEBPα* by MTT assay. Our data confirmed by apoptosis and necrosis assay due to increased apoptosis and necrosis after LNA-anti-miR-182 transfection. The present study results suggested miR-182-5p inhibition can reduction the cell viability predominantly due to induces apoptosis and necrosis through regulation of *CEBPα* gene in APL cell line.

As mention above due to oncogenic role of miR-182-5p and their effect on *CEBPα *as potentially anti-proliferative in myeloid cells, therapeutic strategies using inhibition of miR-182 in APL and other malignant disorders may be useful. The previous studies represented therapeutic strategies by miRNA inhibitors as cancer treatment [35-37]. The LNA is one of the technology as anti-miRNA that used for oncogenic miRNAs inhibition [38]. MiRNAs therapy now reaches the clinical trial stage and promising in cancer treatment. All-Trans Retinoic Acid (ATRA) used for the treatment of APL but a subset of APL patients is not cured with ATRA [39], in this cases maybe combination therapy with LNA-anti-miR and ATRA can cure effect although in vivo studies in the future need to evaluate the feasibility of this strategy. 
